# Scoping review of the literature on factors and interventions to reduce repeat mental health admissions to hospital emergency departments

**DOI:** 10.1192/j.eurpsy.2023.1007

**Published:** 2023-07-19

**Authors:** W. Mao, R. Shalaby, V. Agyapong

**Affiliations:** 1 Psychiatry, University of Alberta, Edmonton; 2 Psychiatry, Dalhousie University, Halifax, Canada

## Abstract

**Introduction:**

Patients with mental health issues visit the emergency department (ED) more often than those with other disorders. Frequently ED visiting not only adversely impacts patients and their families, but also burdens the healthcare system economically. Identifying ways to minimize avoidable ED readmissions has become a hot research topic worldwide.

**Objectives:**

The purpose of this scoping review was to identify influential factors and possible interventions to reduce psychiatric frequent ED visits.

**Methods:**

This scoping review was conducted through a systematic search in major scientific databases, including PubMed, PsycINFO, MEDLINE, JSTOR, Scopus, and Web of Science, to identify factors and interventions contributing to decreasing repeat visits to the emergency department for mental health concerns up to January 2022.

**Results:**

From 6951 publications, 31 articles met the inclusion criteria and were included in this review. This review showed six influential factors and 26 potential interventions were aimed to reduce the ED visits, such as receiving methadone & having a regular family physician, readiness for hospital discharge assessment & perceived coping skills and strategies; The High Alert Program (HAP) & the Patient-Centered Medical Home (PCMH), the Primary Behavioral Health Care Integration (PBHCI) & the Collaborative Care (CC) Program etc.

**Conclusions:**

Worldwide, several initiatives have been taken to reduce ED visits and the associated burden on healthcare systems. Interventions involving comprehensive and multidisciplinary services, incorporating evidence-based behavioral and pharmacological strategies and emphasizing case management were found to be effective. Additionally, there were a marked consideration for diverse mental health groups, such as those with substance use disorder and of young age. This review highlights the greater need for addressing more influential factors, developing accessible interventions, as well as setting up a comprehensive community health care systems aiming to reduce frequent ED presentations.

**Disclosure of Interest:**

None Declared

